# The Central FacilitaTOR: Coordinating Transcription and Translation in Eukaryotes

**DOI:** 10.3390/ijms26072845

**Published:** 2025-03-21

**Authors:** Summer E. Adams-Brown, Ke Zhang Reid

**Affiliations:** Department of Biology, Wake Forest University, Winston-Salem, NC 27109, USA

**Keywords:** TOR, transcription, RNA processing, RNA export, mRNA turnover, translation, stress response, ribosome biogenesis

## Abstract

One of the biggest challenges to eukaryotic gene expression is coordinating transcription in the nucleus and protein synthesis in the cytoplasm. However, little is known about how these major steps in gene expression are connected. The Target of Rapamycin (TOR) signaling pathway is crucial in connecting these critical phases of gene expression. Highly conserved among eukaryotic cells, TOR regulates growth, metabolism, and cellular equilibrium in response to changes in nutrients, energy levels, and stress conditions. This review examines the extensive role of TOR in gene expression regulation. We highlight how TOR is involved in phosphorylation, remodeling chromatin structure, and managing the factors that facilitate transcription and translation. Furthermore, the critical functions of TOR extend to processing RNA, assembling RNA–protein complexes, and managing their export from the nucleus, demonstrating its wide-reaching impact throughout the cell. Our discussion emphasizes the integral roles of TOR in bridging the processes of transcription and translation and explores how it orchestrates these complex cellular processes.

## 1. Introduction

Proper eukaryotic gene expression is an orchestrated process involving multiple stages within the nucleus and cytoplasm. In the nucleus, nascent RNAs such as ribosomal RNAs (rRNAs), transfer RNAs (tRNAs), and messenger RNAs (mRNAs) are transcribed and assembled into ribonucleoprotein particles (RNPs) before being exported to the cytoplasm for protein synthesis. These steps are intricately coordinated to maintain cellular homeostasis. Despite extensive studies on transcription, RNA export, and translation individually, the signaling mechanisms that synchronize these processes remain poorly understood. The Target of Rapamycin (TOR) signaling pathway could be one of the mediators in these complex interactions. This review surveys the components and fundamental roles of the TOR signaling pathway mainly in mammals and two key models of unicellular eukaryotes: budding yeast (*Saccharomyces cerevisiae*, *S. cerevisiae*) and fission yeast (*Schizosaccharomyces pombe*, *S. pombe*) (Table 1). We highlight the evolutionary conservation and divergence of roles of mammalian and yeast TOR complexes to compare their universal and species-specific biological roles. Following this, we provide a detailed overview of how TOR signaling modulates cellular processes at both the transcriptional and translational levels. We explore the mechanisms by which TOR influences gene expression, particularly focusing on its role in promoting the transcription of genes involved in ribosome production and nutrient metabolism. Additionally, we discuss the impact of TOR on translation by regulating the initiation and elongation phases. Finally, we explore the roles of TOR in coordinating transcription and translation, highlighting its essential roles in stress response and ribosome biogenesis. This coordination allows organisms to adapt effectively to environmental changes.

The TOR signaling pathway, highly conserved among eukaryotic cells, is crucial in integrating signals from nutrients such as amino acids, growth factors, and energy resources [1,2]. This integration regulates cellular mechanisms such as metabolism, proliferation, and migration, essential for maintaining cellular homeostasis. The discovery of rapamycin traces back to the 1970s, when it was first found in a bacterial strain, *Streptomyces hygroscopicus*, in a soil sample from Easter Island [3]. This potent antifungal metabolite was isolated and later named after the local name of the island: Rapa Nui. Subsequent research revealed its significant antiproliferative and immunosuppressive effects on mammalian cells, sparking intense scientific interest in understanding the mechanistic action of the drug.

Heitman and colleagues discovered the immunosuppressive properties of rapamycin in 1991 in *S. cerevisiae* [4]. They identified point mutants *tor1-1* (serine 1972 to arginine, Tor1) and *tor2-1* (serine 1975 to isoleucine, Tor2), which exhibited resistance to the growth-inhibitory effects of rapamycin. The same study discovered that the intracellular cofactor FKBP12 (FK506-binding protein 12) is essential for rapamycin-induced toxicity, where the FKBP12–rapamycin complex interacts with Tor1, inhibiting its activity [5]. In following years, R. Loewith and colleagues described two separate and distinct TOR signaling pathways in *S. cerevisiae*: Target of Rapamycin Complex 1 (TORC1) and Target of Rapamycin Complex 2 (TORC2) with the kinases Tor1 and Tor2 the center of these two pathways, respectively [4]. These pathways play a central role in cell proliferation and growth by sensing nutritional status and allowing cell cycle progression from G1 to S phase [6,7]. Later studies led to identifying and cloning the mammalian target of rapamycin, mTOR (mammalian Target of Rapamycin), which, along with its subsequent downstream targets, have emerged as candidates for cancer therapies [8,9].

## 2. TOR Complexes, Components, and General Functions

mTOR, also known as FRAP or RAFT1, is a serine-threonine kinase that belongs to the Phosphoinositide 3-Kinase (PI3K)-related protein kinases family [10]. This enzyme regulates almost all cellular functions that pertain to growth and metabolism in response to exogenous and endogenous stimuli. mTOR is part of two distinct protein complexes, mTORC1 and mTORC2, each characterized by unique structural components (Table 1 and Table 2) [1,2]. mTORC1 includes mTOR, RAPTOR (Regulatory-associated Protein of mTOR, homolog to *Sc*Kog1), and mLST8 (Mammalian Lethal with SEC Thirteen Protein 8, homolog to *Sc*Lst8) [1]. mTORC2 consists of mTOR, RICTOR (Rapamycin-insensitive companion to mTOR, homolog to *Sc*Avo3), mSIN1 (Map Kinase-interacting 1, homolog to *Sc*Avo1), and mLST8 [2]. One mammalian-specific component of the mTORCs is DEPTOR (DEP domain-containing mTOR-interacting protein), which acts as an inhibitor of both mTORC1 and mTORC2 [11], and some cancers are known to overproduce DEPTOR [12]. mTORC1 is more understood than mTORC2 due to its sensitivity to rapamycin, making mTORC1 easy to study in vitro and in vivo. The activation of mTORC1 is highly controlled by upstream signaling pathways that respond to either exogenous growth factors or intracellular nutrient changes and stimulate anabolic pathways to convert energy and nutrients into macromolecules [13]. Regulation of mTORC1 is also mediated by Rag proteins—a family of four related small GTPases, RagA/B/C/D–and their regulators, which localize on the external surface of lysosomes [14,15]. These GTPases function as heterodimers, with RagA/B binding to RagC/D, and their nucleotide-binding capacity is regulated by multi-protein complexes such as RAGULATOR, GATOR1, and GATOR2 [16,17]. Although the precise molecular functions of these protein complexes are still under investigation, early studies show that the presence or absence of amino acids, such as leucine and arginine, can influence these protein complexes [18,19]. Within this amino-acid-induced state, the Rag heterodimer bound to RAGULATOR serves as a docking site for mTORC1 at the lysosome membranes, which then facilitates the activation of mTORC1 by the GTPase Rheb [6,16].

mTORC1 is best known for its role in protein synthesis. It directly phosphorylates Eukaryotic Translation Initiation Factor 4E (eIF4E) binding proteins (4E-BP) [20]. Mammalian 4E-BP is an intrinsically disordered protein (IDP) that exists in three isoforms: 4E-BP1, 4E-BP2, and 4E-BP3 [21]. This phosphorylation of 4E-BP leads to the release of eIF4E, allowing it to bind to eukaryotic translation initiation factors 4G and 4A (eIF4G, eIF4A, respectively), forming the eIF4F translation initiation complex. This complex attaches to the 5′ cap of the mRNA, and recruits the 43S preinitiation complex (PIC), comprising eIF2-GTP, initiator methionyl tRNA (Met-tRNAi), and the small ribosomal subunit (40S). Together, these components assemble into the 48S PIC, ultimately initiating mRNA translation [22]. mTORC1 also influences protein synthesis through the activation of ribosomal S6 protein kinases, S6K1 and S6K2 [23]. Beyond protein synthesis, mTORC1 plays roles in several metabolic processes, including lipid synthesis, aerobic glycolysis, autophagy, lysosome biogenesis, and glutaminolysis [6,24,25].

mTORC2, unlike mTORC1, is not directly inhibited by rapamycin due to its inability to bind with the FKBP12–rapamycin complex [26]. This lack of inhibition has made studying mTORC2 more difficult. As a result, the function and regulation of mTORC2 are less defined than mTORC1, despite mTORC2 being a hub for cellular metabolism involving amino acids, nucleotides, fatty acids, and lipids, like mTORC1 [27]. RICTOR, one of the core components of mTORC2, primarily acts as a scaffolding protein, while mSIN1 likely contains a substrate binding site and determines the localization of mTORC2. Growth factors have been shown to activate mTORC2 in a PI3K-dependent manner [2]. Phosphatidylinositol phosphates, generated by PI3Ks, bind to the pleckstrin homology (PH) domain of mSIN1, which then allosterically relieves its suppressive effects on the kinase domain of mTOR [28]. The identification of mTORC2 effectors has been largely based on genetic studies, and likely, many metabolic roles mediated by mTORC2 are attributed to indirect downstream targets. Notably, mTORC2 phosphorylates AGC kinases, which is a family of serine/threonine kinases including AKT (protein kinase B), protein kinase C family members, and serum- and glucocorticoid-induced kinases 1 (SGK1), at specific conserved motifs within their C-terminal tails, essentially activate them by priming them for further downstream signaling [29,30]. Another downstream effector of mTORC2 is the insulin-like growth factor 2 (IGF2). mTORC2 promotes IGF2 by co-translationally phosphorylating IGF2 mRNA binding protein 1 (IMP1) [31]. Furthermore, mTORC2 can regulate glycolysis and the pentose phosphate pathway (PPP) through phosphorylation of AKT and c-Myc [32]. Specifically, AKT phosphorylation mediated by mTORC2 enhances glucose uptake [33] and glucose-6-phosphate production by hexokinase 2 [34], the first step in glycolysis. While mTORC2 contributes to complex cellular functions related to metabolism, much remains to be uncovered about its broader biological impact [35].

The budding yeast TOR signaling pathway (*Sc*TOR) consists of two structurally and functionally distinct complexes, TORC1 and TORC2, with a rapamycin-sensitive redundant function (Table 1) [4,27]. The essential components of *Sc*TORC1 are Tor1 or Tor2, Kog1, Lst8, and Tco89 [36,37]. In contrast, *Sc*TORC2 is composed of Tor2, Avo1, Avo2, and Avo3 [36,37]. Loss of *Sc*Tor1 has a marginal effect on yeast growth, while loss of *Sc*Tor2 causes lethality and cannot be compensated even with *Sc*Tor1 overexpression [4]. Interestingly, *S. cerevisiae* features two types of TORC1 complexes, each containing either Tor1 or Tor2, while other species, including mammals and *S. pombe*, possess only a single form of TORC1. Despite the belief that these complexes perform similar functions, they exhibit several intriguing assembly properties [38]. Notably, Tor2 is unique in its ability to integrate into both TORC1 and TORC2 complexes, unlike Tor1, which does not participate in TORC2 assembly [36]. Additionally, there are no chimeric TORC1 complexes containing both Tor1 and Tor2; only one or the other is present in any given TORC1 complex [39]. Recent structural engineering efforts have produced a Tor2 mutant incapable of forming TORC1 but still able to assemble into TORC2 [40]. Experimental results with mutant Tor2 strains have shown varied phenotypes compared to the *tor1*Δ strain, influencing factors such as rapamycin sensitivity, caffeine response, pH-dependent growth, and both replicative and chronological lifespans. These findings suggest that the TORC1 subcomplexes may differ in their molecular evolution and impacts on lifespan, highlighting the need for further investigation into their specific functions.

Regulatory factors of budding yeast TORC1 include nutrients, cellular energy, and growth factors. Specifically, amino acid abundance regulates TORC1 through mechanisms involving the RAG family of small GTPases, Gtr1 and Gtr2, in budding yeast [41]. The EGO/GSE ternary complex (composed of Ego1, Ego2, and Ego3), the ortholog of RAGULATOR, tethers Gtr1 and Gtr2 GTPases to the vacuole and facilitates their dimerization [42,43], thus activating TORC1. Additionally, the SEACIT and SEACAT complexes are the budding yeast counterparts of GATOR1 and GATOR2, respectively [44,45]. The active Gtr1^ATP^–Gtr2^ADP^ binds to Kog1 to stimulate TORC1. However, the precise molecular details of this interaction remain elusive, with suggestions that the underlying mechanism may differ from those in mammals [39,46]. TORC2 is localized in the plasma membrane and directly responds to stressors by phosphorylating protein kinases PKC1 and Ypk2 [47,48]. PKC1 is essential for cell wall remodeling during growth, analogous to the alpha-, beta-, and gamma isoforms of mammalian PKC [49]. Ypk2 regulates ceramide synthesis, which serves as substrates for plasma membrane lipid biosynthesis, an essential process for cell growth [50]. Additionally, TORC2 has a unique, rapamycin-insensitive role in actin cytoskeleton reorganization, first identified in yeast but later found in other higher eukaryotes, including humans [17,51,52].

It has been shown that cells lacking *Sc*TORC1 function, either through rapamycin treatment or depletion of Tor1, arrest growth and rapidly exhibit characteristics of the stationary (G0) phase, such as reduced translation initiation. This loss of TORC1 activity particularly affects the cell cycle by altering the translational control of the G1 cyclin, Cln1 [53,54]. Further studies report that eIF4G, an essential initiation factor required for mRNA translation, is rapidly degraded upon entry of the diauxic growth phase, which is the transition from fermentative to oxidative metabolism in *S. cerevisiae* or with rapamycin treatment [55]. This degradation of eIF4G was not limited to a specific stage of the cell cycle, and the degradation pathway of eIF4G is unknown, but it is known to be initiated upon rapamycin treatment [55].

In the fission yeast *S. pombe*, the TOR signaling pathway is a critical regulator of cellular processes. *Sp*TOR Complex 1 (TORC1), comprising the catalytic subunit *Sp*Tor2, Mip1 (the RAPTOR ortholog, also known as Kog1), Wat1/Pop3 (the mLST8 ortholog), and Tco89, is essential for cell growth (Table 1 and Table 2) [56]. Overproduction of Tor2 leads to inhibited sexual differentiation, mating, and meiosis. Meanwhile, disruption of TORC1 simulates nitrogen starvation and triggers G1 phase cell growth arrest, characterized by small, rounded cells, induction of nitrogen starvation-specific genes, and suppression of sexual development [57]. Like mammals, *S. pombe* Rheb GTPase Rhb1 is an essential activator of TORC1 [58,59,60]. RAGULATOR- and GATOR1-like complexes have also been identified in *S. pombe* [61]. Similar to their mammalian counterparts, these complexes regulate the cellular localization and nucleotide-binding state of Gtr1–Gtr2, respectively. However, the activation of *Sp*TORC1 does not depend on Rag-like GTPases; instead, these GTPases play a crucial role in modulating TORC1 activity on vacuolar membranes, ensuring an optimal cellular response to nutrients [61]. *Sp*TORC1 is influenced by various inputs, including nitrogen, carbon sources, internal and external stressors, energy levels, and oxygen availability [62]. Collectively, these impact the regulation of gene expression and metabolic processes, thereby shaping cellular phenotypes [63].

In contrast, *S. pombe* TOR Complex 2 (TORC2) includes *Sp*Tor1 as the catalytic subunit, along with Ste20 (the RICTOR ortholog), Sin1 (the mSIN1/Avo1 ortholog), Wat1/Pop3, and Bit61 (Table 1) [56,64]. Although *Sp*Tor1 is not essential, its association with TORC2 highlights its multiple roles in stress response, G1 arrest, gene silencing, telomere integrity, and sexual development [62,65,66]. *Sp*TORC2 is crucial for timing the G2/M transition, enhancing stress survival, and facilitating sexual development through phosphorylation and activation of AGC kinase Gad8 [67,68]. Furthermore, the activity of *Sp*TORC2 is modulated by glucose levels, osmotic stress, and cellular quiescence [69]. Both *Sp*TORC2 and *Sc*TORC2 play roles in conferring resistance to genotoxic stress and reducing DNA damage foci accumulation [70]. The precise mechanisms by which *Sp*TORC2, through Gad8, manage genome integrity and DNA damage resistance are yet to be fully elucidated [68].

**Table 2:** Essential players of TOR complexes and their general functions.

mTOR/*Sc*TOR/*Sp*TOR	Function	Source(s)
mTOR Tor1 and Tor2 Tor1 and Tor2	Serine/threonine kinase Catalytic subunit of TOR complex(s)	[1,2,13,71,72]
RAPTOR Kog1 Mip1	Adapter/scaffolding protein of the TOR complex(s), might have a role in recruiting and binding substrates of TORC1, and interacts with Rag GTPases	[73,74,75]
mSIN1 Avo1 Sin1	Scaffolding protein important in the integrity of (m)TORC2	[76,77,78,79]
mLST8 Lst8 Wat1	WD repeat protein, protein scaffolding and structural integrity of TORCs	[80,81]
RICTOR Avo3 Ste20	Activates protein-kinase B (AKT) signaling	[64,82,83]

## 3. TOR Signaling in the Regulation of Transcription, Translation, and Intermediate Steps of Gene Expression

**Figure 1:**
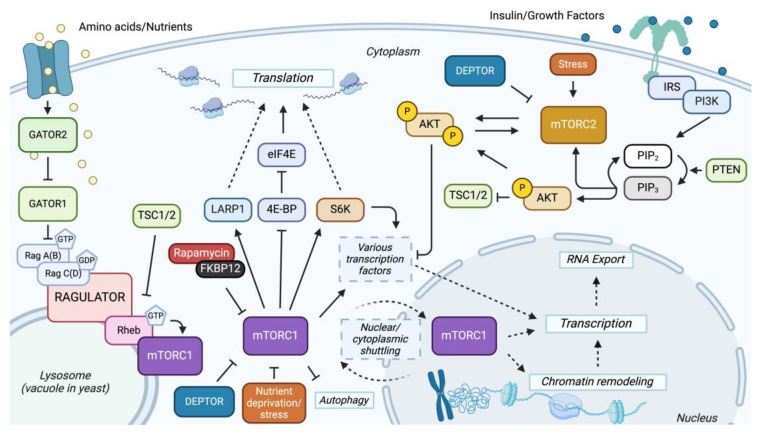
Overview of mTOR signaling pathway: The mTOR signaling network consists of two branches, each mediated by a specific complex, mTORC1 and mTORC2. The GATOR1 complex inhibits rapamycin-sensitive mTOR by acting as a GTPase-activating protein for Rag GTPase and, therefore, inhibits mTORC1 by converting the active GTP-bound Rag proteins to inactive GDP-bound forms. GATOR2 is a positive regulator of mTORC1 by inhibiting GATOR1 [15]. Once mTORC1 is activated, it leads to the phosphorylation of LARP1, S6Ks, and 4E-BP, promoting translation [86,87]. Activated mTORC1 also leads to the activation of transcription factors and nuclear/cytoplasmic shuttling of the complex, leading to the upregulation of genes involved in nucleotide biosynthesis, lipid biosynthesis, ribosomal biosynthesis, hypoxia, and others [27,86]. Conversely, activated mTORC2 regulates cell survival, metabolism, and cytoskeletal dynamics by directly phosphorylating AKT (protein kinase B) and others [27,88]. The energy-sensing PI3K-PTEN-AKT-TSC pathway also activates AKT and is a positive regulator of mTORC1 by inhibiting the TSC1/2 (Tuberous Sclerosis) complex [88]. The TSC complex inhibits mTORC1 by serving as a GTPase-activating protein for the Ras homolog Rheb, a small GTPase. Rheb contributes to the activation of mTORC1 by promoting lysosomal surface translocation [16]. Solid lines indicate a direct interaction, and dotted lines indicate an indirect interaction, either from an unknown mechanism or other effectors.

Unlike in prokaryotes, eukaryotic transcription and translation are spatially separated by the nuclear membrane, with transcription occurring in the nucleus and translation in the cytoplasm. This separation allows for more complex regulation. mRNA transcripts are matured in the nucleus before being exported to the cytoplasm through nuclear pores, ensuring only properly processed mRNAs are translated. Several critical signaling pathways govern the coordination between transcription and translation, connecting gene expression at multiple levels to ensure proteins are synthesized according to cellular needs [34,84,85]. The TOR signaling pathway plays a vital role in this regulatory network, integrating environmental cues (Figure 1). This integration enables the TOR signaling pathway to finely tune transcriptional and translational processes, aligning protein synthesis with cellular homeostasis in various conditions.

### 3.1. TOR in Transcription by Modulating Transcription Factors and Chromatin Structure

mTOR signaling has been shown to modulate the expression of transcription factors (TFs) via translation of their related mRNA, cellular localization, activity via direct phosphorylation, stability via modulation of the ubiquitin/proteasome system (UPS), the regulation of accessory factors, or in combination of one or more of these factors [71,89]. TOR complexes also compartmentalize to various subcellular locations, such as the nucleus, cytoplasm, and ribosomes, where they can directly modulate the activity of TFs, ribosomal proteins, and translation factors [6]. TOR signaling adjusts the activity of key TFs to control the expression of genes critical for growth and metabolism. For instance, mTORC1 signaling enhances lipid synthesis by upregulating multiple lipogenic genes [90,91,92,93]. A vital group of transcription factors in lipid synthesis is the sterol regulatory element-binding proteins (SREBPs), including SREBP1a, 1c, and 2 [90]. Portsmann and colleagues initially discovered that rapamycin disrupts the nuclear accumulation of SREBPs and reduces the expression of lipogenic genes, suggesting that mTORC1 facilitates the trafficking, processing, and transcriptional activity of SREBPs [24].

mTORC1 can alter the activities of TFs via direct phosphorylation. For example, activated mTORC1 phosphorylates S6K1, which controls the transcription of certain ribosomal protein genes [94]. S6K1 phosphorylates the cAMP response element binding protein (CREB) isoform, CREMτ, and UBF-1, a nucleolar transcription factor [95]. Phosphorylation of UBF-1 activates RNA Pol I-mediated transcription of genes that encode for rRNAs, thus linking this protein kinase to the upregulation of ribosomal protein RNAs both on the transcriptional and translational level, jointly mediated by mTOR. Orthologs of S6K1 exist in both *S. cerevisiae* and *S. pombe*: Ypk3 and Psk1, respectively [96,97], but less is known about these proteins outside their protein kinase activity. In a recent study, Ypk3 was affinity-captured by Transcription Factor II-B [98], which could suggest that these two proteins have a functional connection.

In *S. cerevisiae*, the TORC1 signaling pathway can influence transcription through the GATA transcription factor Gln3 [99]. Gln3 is a transcriptional activator within the nitrogen catabolite repression (NCR) system. Under normal conditions, TORC1 phosphorylates Gln3 through Tap42-dependent inactivation of Sit4, a type 2A-related serine-threonine phosphatase, leading to the retention of Gln3 in the cytosol [100]. However, when TORC1 is inactivated, either due to nutrient starvation or rapamycin treatment, the Tap42–Sit4 complex dissociates. This allows Sit4, along with PP2A, to dephosphorylate Gln3 either directly or indirectly [101]. Subsequently, Gln3 can enter the nucleus to upregulate genes essential for nitrogen metabolism and its regulation.

Additionally, TOR signaling is implicated in altering chromatin structure that affects transcription. mTOR can interact with chromatin components, and its direct recruitment to chromatin appears responsive to the mTOR inhibitors rapamycin and Torin-1 [102,103,104]. ChIP-seq analyses of mouse liver revealed that mTOR binds to the regulatory regions of genes genome-wide, including enrichment at genes involved in the citric acid cycle and lipid biosynthesis [105]. Dufour and colleagues recently uncovered several chromatin-bound mTOR interactors in prostate cancer cells [102]. These interactors include diverse chromatin remodeling complexes such as NURD (Nucleosome Remodeling and Deacetylase), CoREST (Corepressor for REST), BHC (BRAF–HDAC complex), the SWI/SNF BAF complex, and NcoR/SMRT (Nuclear receptor co-Repressor/silencing mediator of retinoid and thyroid hormone receptor) along with several transcription factors and proteins involved in histone methylation, DNA repair, and protein SUMOylation. Similarly, in *S. pombe*, the TOR components Gad8 and Tor1 are identified within the chromatin-bound fraction in the nucleus [106]. *Sp*Tor2 targets a conserved nuclear RNA elimination system, particularly the serine- and proline-rich protein Pir1, to control gene expression through RNA decay and facultative heterochromatin assembly [107]. Fission yeast TORC2 was also essential for chromatin-mediated gene silencing and subtelomeric heterochromatin assembly [108]. These mechanisms are conserved from fission yeast to higher eukaryotes but are missing in *S. cerevisiae*, which lacks large heterochromatic domains. Altogether, these findings underscore nuclear TOR as a key chromatin regulator, thereby affecting the accessibility of transcriptional machinery to DNA [109].

### 3.2. TOR in Alternative Splicing

During and after transcription, precursor mRNA (pre-mRNA) undergoes several processing steps, including capping, splicing, and polyadenylation. Pre-mRNA splicing is a critical step in determining what proteins are made by a cell, and when this process is disrupted, it can contribute to the development of several age-related chronic diseases [110,111]. Many studies have shown that TOR signaling is critical in controlling alternative splicing in various organisms. Heintz and colleagues used transcriptomics and splicing analysis in *C. elegans* to investigate age-related changes in pre-mRNA splicing under ad libitum feeding and dietary restriction. The authors found that splicing defects accumulate with age but are mitigated by dietary restriction through splicing factor 1 (SFA-1), the *C. elegans* homolog of SF1 [111]. SFA-1 plays a crucial role in extending lifespan when the animal is put on a dietary restriction diet, and this effect is mediated through its influence on the TORC1 signaling pathway, specifically by interacting with components like AMPK, RAGA-1, and RSKS-1/S6 kinase [111].

Results from a recent genetic screening in *C. elegans* indicate that nutrient-induced TORC1 regulates broad alternative mRNA splicing during larval growth [112]. This TORC1-mediated regulation of mRNA splicing and the generation of mRNA isoforms are largely independent of S6K, instead, via enhancing the activity of the serine/arginine-rich (SR) protein RSP-6 (SRSF3/7) and other splicing factors. In agreement with this study, mTORC1 hyperactivation is related to elevated exon skipping in mammalian cells, which is partially attributed to increased SRSF3 [113]. In *S. cerevisiae*, TOR kinase functions are essential to upregulate the translational derepression of *HAC1* mRNA in response to cellular stress via nonconventional cytosolic splicing [110]. These findings demonstrate that TORC1 controls the bulk of gene expression regulation events during nutrient response, including a widespread remodeling of alternative mRNA splicing.

### 3.3. TOR in RNA Export

Newly transcribed RNAs, such as rRNAs and mRNAs, are assembled into ribonucleoprotein particles (RNPs) and must bind to export factors to be exported from the nucleus to the cytoplasm for protein synthesis. This export is critical for maintaining cellular homeostasis, yet the regulation of this process remains poorly understood [114]. Inhibition of the PI3 kinase/AKT pathway increases cytoplasmic and reduces nuclear levels of poly(A) RNA [114], suggesting a potential exacerbation of mTORC1 function when PI3K or AKT is inhibited. This result could indicate that mTORC2 may disrupt RNP export via the PI3K/AKT pathway, although the exact mechanisms remain to be elucidated. Furthermore, TOR signaling is implicated in regulating the subcellular distribution of proteins essential for pre-40S ribosome export and cotranscriptional ribosome assembly. A study in *S. cerevisiae* demonstrated that the ribosome synthesis factors Dim2 and Rrp12 accumulate in the nucleolus under stress in a TOR-dependent manner [115]. The full scope of the involvement of TOR signaling in RNA export is unclear, highlighting a significant gap in our understanding of TOR in this critical intermediary step between transcription and translation.

### 3.4. TOR in mRNA Turnover

The stability and turnover rates of mRNAs play crucial roles in regulating gene expression, as they determine how long an mRNA is available for translation. mRNA stabilities are influenced by sequences within the mRNA, such as the length of the poly(A) tail, and by proteins that bind to these sequences. Evidence provided from *S. cerevisiae* supports that the TOR signaling pathway controls mRNA turnover. Albig and Decker showed that a group of mRNAs was destabilized during nutrient limitation or after rapamycin treatment [116]. They also found that inhibiting the TOR pathway accelerated the major mRNA decay mechanism in yeast, the deadenylation-dependent decapping pathway. Rapidly responding mRNAs are destabilized because of prematurely short poly(A) tails, resulting from either rapid deadenylation or destabilized polyadenylation. Conversely, slowly responding mRNAs undergo destabilization primarily through rapid decapping [89]. In mammals, the 3′ untranslated region (3′ UTR) size regulation through alternative polyadenylation is increasingly associated with cell metabolism [117]. However, whether yeasts and metazoans share similar mechanisms in energy-mediated alternative polyadenylation regulation remains an ongoing examination.

### 3.5. TOR in Translation

Protein synthesis is an energy-intensive process, so TOR signaling tightly and immediately regulates mRNA translation [12,86]. This regulation mainly affects translation initiation [86]. Two of the best-known targets of TOR signaling that directly promote protein synthesis are the S6K1 [118] and 4E-BP1/2 [119]. Without signaling from mTOR, S6K1 binds to eIF3, allowing inactive S6K1 to be tethered to the PIC; upon mTOR activation and subsequent phosphorylation of S6K1, this interaction weakens, leading to S6K1 dissociation and activation, enabling it to phosphorylate other translational targets and promote translation initiation [86,118]. Upon activation, S6K1 targets several key factors essential for the regulation of translation. For example, it directly phosphorylates Rps6 (small ribosomal subunit protein eS6) [23]. Phosphorylation of Rps6 is correlated with mitogenic stimulation and upregulated translation initiation of mRNAs containing 5′-terminal oligopyrimidine (5′-TOP) tracts, which indicates that they encode for components of protein synthesis machinery [120]. S6K1 also phosphorylates and activates eIF4B, which is recruited to eIF4A and facilitates its helicase activity in unwinding the secondary structure in the 5′ untranslated region (5′ UTR) of mRNAs [118]. Additionally, S6K1 plays a role in feedback regulation of mTORC2 by mTORC1 by phosphorylating RICTOR, inhibiting mTORC2 and thus AKT signaling. S6K1 also phosphorylates DEPTOR, which helps regulate both mTORC1 and mTORC2 [121]. This phosphorylation event is thought to alter the ribosome structure, making it more likely to bind to 5′-TOPs [122].

Activated mTORC1 phosphorylates 4E-BP1 on sites that regulate the interaction with the 5′ cap-binding protein of eIF4E, leading to eIF4F complex formation. A two-step model has been proposed for 4E-BP1 phosphorylation [20]. mTOR first phosphorylates Thr37 and Thr46 on 4E-BP1 complexed with eIF4E. The second step is the phosphorylation at Ser65 and Thr70 of the C-terminus of 4E-BP1 [20,21]. Through these phosphorylation events, mediated by mTORC1, eIF4E can bind to translation initiation factors 4G and 4A to form the eIF4F initiation complex that binds to the 5′ cap of mRNA. From there, the eIF4F complex interacts with the eIF2 ternary complex, where it can recruit the 40S ribosomal subunit and scan the mRNA until a start codon is reached. The mechanism by which TORC1-4E-BP governs translation initiation is conserved in yeast, with two yeast 4E-BPs acting as substrates of TORC1 [123].

Remarkably, TOR signaling helps determine which mRNAs are preferentially translated under different conditions. Previous studies using ribosome profiling revealed that nearly all mRNAs (99.8%) show translational inhibition after treatment with the mTOR inhibitor Torin-1, with a general reduction in translational efficiency by about 50% [124,125]. Some mRNAs, primarily those involved in protein synthesis and containing 5′-TOP motifs, are more significantly affected [124,126]. In cells lacking both 4E-BP1 and 4E-BP2, Torin-1 has little effect on these mRNA translations, suggesting that the regulation of these mRNAs largely depends on 4E-BP [124]. Two models have been proposed to explain the selective regulatory role of 4E-BP in translation. The first model suggests that 4E-BP binds solely to eIF4E, inhibiting the formation of the crucial eIF4F complex and thereby reducing eIF4E-dependent mRNA translation. The second model posits that 4E-BP not only binds to eIF4E but also associates with mRNAs, directly inhibiting their translation. These models can coexist and are not mutually exclusive, and in vitro and in vivo evidence exists to support both [86,127,128,129].

Besides 4E-BP, La-related protein 1 (LARP1) contributes to translation regulation in the mTOR pathway. LARP1 is a large RNA-binding protein containing an eIF4G-like motif, RNA-biding La motif (LAM), RNA recognition motif-like (RRM-L), and a unique DM15 region in the C-terminus [125,130,131]. It plays diverse roles in regulating 5′-TOP mRNA translation [87,125,130,132,133] and in mRNA stability [87,132,134,135]. In conditions where mTOR is active, LARP1 enhances general mRNA translation and has a more pronounced effect on 5′-TOP mRNA translation [136]. When mTOR is inactivated, LARP1 binds to the 5′ cap and 5′-TOP regions of transcripts and functions as a repressor of TOP mRNA translation [132,133].

Based on these findings, Yang and colleagues proposed a new model to explain the preferential translational control by the mTOR pathway [71]. Upon mTORC1 activation, phosphorylated 4E-BP becomes inactive. Concurrently, LARP1 is phosphorylated by mTORC1 and AKT/S6K1, and it binds to the 3′ UTRs of its target mRNAs, including 5′-TOP mRNAs, to enhance their translation [87,137]. When mTORC1 is inactivated, hypo-phosphorylated LARP1 binds directly to the 5′ caps, 5′ TOP sequences, and 3′ UTRs of TOP mRNAs, inhibiting their translation [132,133]. At the same time, the hypo-phosphorylated form of 4E-BP attaches to eIF4E and the mRNA 5′ cap regions, preventing the formation of the eIF4F complex. This may result in a competition between 4E-BP-eIF4E and LARP1 for binding to the 5′ TOP mRNAs, potentially ensuring complete translational suppression of these mRNAs and recycling of cellular components, helping the cells conserve energy.

## 4. TORC1 and TORC2 Signaling in Cellular Stress Response Pathways

Stress signals can coordinate transcription and translation through various mechanisms, enabling an organism to adapt and respond effectively to environmental changes. When nutrients are scarce, TORC1 activity decreases, reducing phosphorylation of the protein synthesis machinery, such as S6K1 and 4E-BP, thereby reducing their interaction [138]. This results in a general slowdown of protein synthesis as the translation initiation process becomes less efficient. This shift helps save energy, provides essential building blocks and energy through recycling cellular components, and focuses on producing stress response proteins. For example, *Sc*TORC1 signaling can modulate the localization and activities of stress-inducible transcription factors, Msn2/Msn4, which bind and activate genes containing the stress response element in response to a wide variety of stresses [99,139,140,141,142]. The *Sc*TOR-controlled transcription factors, Gln3 and Rtg1/Rtg3, which mediate glutamine synthesis, are activated under glutamine starvation [106,142].

The Integrated Stress Response (ISR) coordinates the downregulation of general protein synthesis while selectively allowing the translation of specific proteins crucial for stress recovery. The ISR is triggered by activating one of several stress-sensing protein kinases that detect different types of cellular stress [143]. There are four eIF2α kinases in mammals, GCN2 (general control non-derepressible 2), PERK (PKR-like endoplasmic reticulum kinase), PKR (protein kinase R), and HRI (heme-regulated inhibitor) [143]. Three exist in fission yeast, Gcn2, Hri1, and Hri2, and one in budding yeast, Gcn2 [144,145]. Different types of cellular stress activate each of these kinases: GCN2 is activated by amino acid deprivation [146,147]; HRI is typically activated by heme deficiency [148]; and PERK is activated in response to ER stress caused by the accumulation of misfolded proteins in the ER [149,150,151]. Each of these kinases phosphorylates eIF2α. Phosphorylation of eIF2α is a critical step in the ISR as it leads to a rapid decrease in general protein synthesis, which helps conserve resources during stress.

Although TOR and ISR pathways are essential for cellular adaptation to various stress conditions, they operate through somewhat different mechanisms and have distinct yet intersecting roles. TOR signaling may intersect with the ISR primarily through TORC1, which can be inhibited by stress conditions that activate the ISR [152]. However, little is known about how these two pathways overlap. Some evidence supports that TOR and ISR signaling pathways are coordinated in response to diet and drug-mediated forms of amino acid deprivation [153,154,155,156]. It has also been shown that the ISR and mTORC1 are activated in mouse liver administered HF (halofunginine), a small molecule inhibitor of glutamyl-prolyl-tRNA synthetase (EPRS) [153,157]. HF treatment induces ISR-directed translational control by mimicking amino acid depletion via accumulation of uncharged tRNAs that activate GCN2. HF-treated cells elevate mTORC1 activity. Without GCN2, mice fail to suppress mTORC1 upon HF treatment [153]. In addition, other studies revealed that under conditions of amino acid deprivation, activated GCN2 enhances ATF4 expression, which in turn induces the production of the stress response protein Sestrin2, essential for maintaining the repression of mTORC1 by preventing its localization to the lysosomal membrane [154,155]. Moreover, in *S. cerevisiae*, TORC1 prevents the phosphorylation of Gcn2 through Tap42, a regulator of type 2A-related phosphatase [158], whereas *S. pombe* Tor2 prevents Gcn2 activation in the presence of nitrogen and amino acids [159]. Furthermore, prolonged inhibition of protein synthesis causes a rapid reduction in REDD1 protein, a known mTORC1 inhibitor regulated in development and DNA damage response, resulting in activation of mTORC1 activity [160]. The activated mTORC1 may help to coordinate stress recovery and facilitate the resumption of growth and proliferation by ramping up anabolic processes and reducing catabolic processes.

Studies in *S. pombe* support the notion that stress signals can directly activate mTORC2 activity [27]. The genes involved in the TORC2-AKT signaling pathway in *S. pombe* were initially identified as essential for cell cycle arrest at the G1 phase in response to low nitrogen stress [65,161,162,163]. Under nitrogen starvation conditions, wild-type *S. pombe* cells undergo G1 cell cycle arrest before entering the sexual reproduction phase. The TORC2 signaling pathway is crucial for initiating these sexual processes, and a deficiency in TORC2 results in sterility in fission yeast. These findings indicate the necessity of the TORC2 pathway in enabling *S. pombe* cells to manage low nitrogen stress. This is further evidenced by the role of TORC2 in the transcriptional regulation of *isp7^+^*, a gene encoding an amino acid transporter protein during nitrogen starvation [164].

In addition to responding to nitrogen starvation, TORC2 regulates glucose metabolism and supports cell proliferation under conditions of low glucose stress [69]. Under these glucose-limited conditions, the adenosine monophosphate (AMP)-activated protein kinase (AMPK) signaling pathway is essential for cell growth [165]. Specifically, *S. pombe* cells with mutations impairing either the TORC2 or AMPK pathways cannot form colonies on glucose-limited media. A key player in this process is Ght5, a high-affinity hexose transporter crucial for cell growth when glucose is scarce. The AMPK signaling pathway is necessary for the transcriptional upregulation of *ght5^+^* in response to glucose limitation, while the TORC2 pathway is critical for the proper localization of Ght5 on the plasma membrane [165,166]. Cells deficient in TORC2 show impaired proliferation due to the abnormal degradation of Ght5 in vacuoles, leading to decreased glucose uptake under glucose-limited conditions. The persistence of Ght5 on the plasma membrane is facilitated through α-arrestin, which is regulated by TORC2 [69]. This regulatory mechanism is conserved across species, as evidenced by the arrestin-mediated internalization of hexose transporters in mammalian cells regulated by AKT [167,168].

Under stress, mTOR signaling also regulates protein catabolism, most notably autophagy, which is a process that breaks down and recycles old or damaged parts of a cell. Autophagy is a crucial cellular process triggered by various forms of stress, including starvation, oxidative stress, and infection [169,170,171]. This adaptive response enables cells to maintain homeostasis by degrading damaged organelles and misfolded proteins, recycling essential nutrients, and eliminating potential sources of further cellular stress. The regulation of autophagy is complex and involves multiple signaling pathways. TORC1 acts as a negative regulator of autophagy [172]. Under stress conditions, particularly nutrient deprivation, the inhibition of TORC1 is a key trigger that activates autophagic machinery. This inhibition removes the suppressive impact that TORC1 has on the early stages of autophagy. As a result, the activated initiation complex facilitates the nucleation and assembly of autophagosomes—vesicles that encapsulate and transport cellular components to the lysosome for degradation and recycling [169,170,171]. In contrast to TORC1, the TORC2 signaling pathway plays a supportive role in autophagy under specific conditions. For example, during periods of amino acid scarcity, *Sc*TORC2, through its target kinase Ypk1, acts as a positive regulator of autophagy flux [173]. Ypk1 is instrumental in adapting cell membrane composition and signaling to the stressed state, facilitating the continuation of autophagy. This modulation ensures that autophagy remains efficient and responsive to the cellular environment, aiding in the survival of the cell during prolonged periods of nutrient limitation [173,174].

## 5. TOR Signaling Can Coordinate Transcription and Translation by Regulating Ribosome Biogenesis

**Figure 2:**
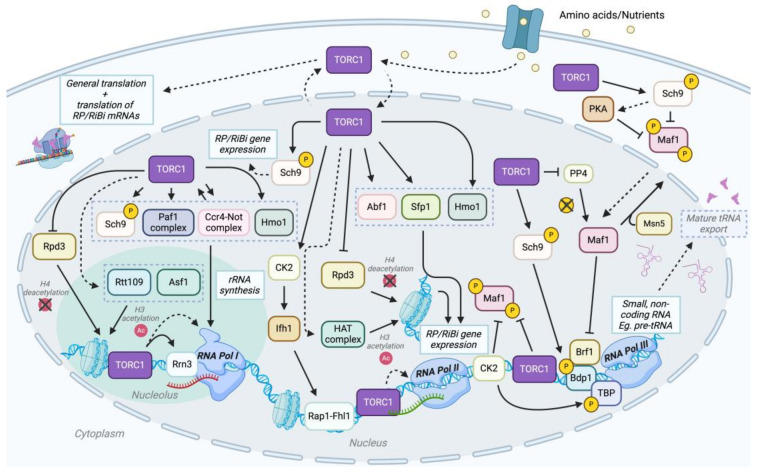
TORC1 can coordinate transcription and translation by regulating ribosome biogenesis in *S. cerevisiae*. TORC1 plays a pivotal role in ribosomal protein (RP) and ribosomal biogenesis (RiBi) gene expression by regulating RNA Pol I, II, and III through direct and indirect mechanisms and various cofactors [71]. In RNA Pol I-mediated transcription, under optimal, nutrient-rich conditions, TORC1 localizes to the nucleus and associates with chromatin, promoting histone modifications such as H3 acetylation via Rtt109 and Asf1 and H4 deacetylation via Rpd3 [104,181]. TORC1 also facilitates Rrn3-RNA Pol I association and further regulates transcription via Sch9, Paf1 complex, Ccr4-Not complex, Hmo1, and others [71]. TORC1 also contributes to ribosome biogenesis and ribosomal protein production by phosphorylating Sch9, which phosphorylates RiBi/RP repressors, activating RiBi/RP gene expression [182]. Ifh1 is phosphorylated by Casein Kinase 2 (CK2), a downstream effector of TOR, and interacts with Rap1-Fhl1 to drive RP/RiBi gene expression [183]. Other TORC1-dependent factors involved in RNA Pol II transcription include Hmo1, Abf1, and Sfp1 [183]. Lastly, TORC1 facilitates RNA Pol III-mediated transcription of small non-coding RNAs such as tRNAs by phosphorylating Sch9, thus activating TFIIIC subunit Bdp1 [184,185]. TORC1 also phosphorylates Maf1 on chromatin, like CK2, thus preventing Maf1 from interacting with RNA Pol III and modulating Maf1 nuclear export via Msn5 [184]. TORC1 integrates nutrient signaling with transcriptional regulation across all three RNA polymerases to support ribosome biogenesis and cellular growth. Additionally, cytoplasmic TORC1 plays a role in modulating RP/RiBi mRNA translation. Solid lines indicate a direct interaction, and dotted lines indicate an indirect interaction, either from an unknown mechanism or other effectors. Proteins within gray dotted boxes (such as Sch9, Paf1 complex, Ccr4-Not complex, Hmo1; Abf1, Sfp1, Hmo1) indicate that all listed proteins have similar downstream effects, specifically rRNA synthesis and RP/RiBi gene expression, respectively.

TOR signaling is an essential regulator of ribosome biogenesis, a complex and essential process spanning both the nucleus and cytoplasm [175,176]. Ribosome biogenesis initiates in the nucleolus, where RNA Pol I transcribes rRNA from ribosome DNA (rDNA) genes arrayed in tandem on chromosomes. These long strands of pre-rRNA undergo a sequence of chemical modifications, cleavages, and trimmings by nucleolar proteins. Meanwhile, RNA Pol II transcribes the genes for ribosomal proteins, which are translated into the cytoplasm and then transported back to the nucleolus. In the nucleolus, they sequentially bind to the pre-rRNA, aiding the modification efforts of the nucleolar proteins. These pre-ribosomes are subsequently exported to the nucleus, where they undergo additional modifications before being dispatched to the cytoplasm as two subunits, 40S and 60S. The final maturation steps occur in the cytoplasm: the 43S PIC, comprised eIF2-GTP-Met-tRNAi ternary complex, initiation factors eIF1, eIF1A, eIF3, eIF5, and the small ribosomal subunit (40S), is recruited to the cap-binding complex eIF4F at the 5′ end of mRNA to form the 48S PIC and begins scanning for a Kozak sequence [22]. This scanning process leads to recognizing a start codon by the 48S PIC, releasing most initiation factors. The subsequent binding of eIF5B promotes the joining of the 60S large subunit, resulting in the last major step of translation initiation, the formation of the 80S initiation complex (80S PIC) [22]. This intricate process is finely tuned to match the cellular metabolic state and external environmental signals. TOR signaling enhances this coordination by stimulating RNA Pol I, II, and III, boosting the transcription of rRNAs, tRNAs, and ribosomal protein genes [71,177]. It also promotes nucleotide biogenesis and the processing and assembly of rRNA [178,179], thus ensuring efficient ribosome construction. Furthermore, TOR signaling facilitates the translation of ribosomal protein mRNAs, completing the mechanisms that support robust protein synthesis and cellular growth [180]. Here, we discuss the role of TOR signaling in synchronizing the activities of the three nuclear RNA polymerases during ribosome biogenesis (Figure 2), which has become a focal point of research, highlighting its critical function in cellular regulation.

### 5.1. RNA Pol I

The production of rRNA, transcribed by RNA Pol I, represents nearly 60% of all transcription in *S. cerevisiae* and is the rate-limiting step of ribosomal biogenesis [186]. It is well established that the inhibition of TORC1 by rapamycin treatment results in severe depletion of RNA Pol I transcription [71]. The phosphorylation of RNA Pol I is crucial for transcription initiation with 115 different sites of phosphorylation, or phosphosites, across all 14 RNA Pol I subunits [187,188]. Although RNA Pol I could be a direct target of TORC1, it has not been described. The mechanisms by which TORC1 regulates RNA Pol I transcription remain a topic of ongoing debate. The current model suggests that TORC1 primarily regulates this process through the transcription factor Rrn3 [71], with rapamycin disrupting the association between Rrn3 and RNA Pol I. Rrn3 also functions as a bridge between RNA Pol I and other transcription factors essential for rDNA transcription [189]. Additionally, RNA Pol I recruitment to rDNA is mediated by the budding yeast ortholog of S6K, Sch9, and, to a lesser extent, Tap42, both of which are known targets of TOR signaling [190].

Other factors reported to influence RNA Pol I transcriptional activity via TOR signaling include the high mobility group B (HMG) family member Hmo1, the Ccr4–Not complex, and the Paf1 complex [71]. Hmo1 localizes close to the nucleolus [191] and rDNA genes [192] and dissociates from rDNA promoters with rapamycin treatment, and its expression is regulated in a TOR-dependent manner [71]. In *S. cerevisiae*, the Ccr4–Not complex is associated with both RNA Pol I and rDNA, indicating this complex has a role in the initiation, elongation, and regulation of this polymerase [193]. Under nutrient-rich conditions, the Ccr4–Not complex inhibits RNA Pol I initiation by modulating its interactions with Rrn3. Conversely, disruption of Ccr4–Not impedes the reduction of RNA Pol I transcription typically observed when TORC1 is inhibited. Additionally, disruption of this complex prevents Rrn3 from dissociating from RNA Pol I following TORC1 inactivation, suggesting Ccr4–Not bridges TORC1 signaling with RNA Pol I regulation [71,193].

The Paf1 complex (Paf1C), like the Ccr4-Not complex, is a well-characterized transcription elongation factor mainly known for its roles in histone modifications, RNA processing, and export [194,195,196]. In vitro analyses demonstrated that Paf1C can increase the rate of transcription elongation of RNA Pol I on a blank rDNA template. This finding indicates that Paf1C could directly bind to the polymerase to enhance elongation kinetics [197]. Curiously, overall transcription was not affected in *paf1*Δ cells upon histidine starvation, and rapamycin only partially causes RNA Pol I transcription reduction. These results indicate that Paf1C is not only a factor that regulates RNA Pol I transcription under optimal growth conditions but also plays a role in its repression when TORC1 is inhibited [197].

Additionally, TOR signaling plays a significant role in mediating epigenetic and chromatin changes that regulate RNA Pol I activity. For example, several histone H3 modifications are positively regulated by TORC1, including H3 lysine 56 acetylation (H3K56ac). This modification is well known for regulating DNA replication, repairing damage, and maintaining genomic stability by facilitating proper nucleosome assembly on newly synthesized DNA [198]. Inhibition of TOR decreases H3K56ac by reducing the association of Hmo1 with rDNA, thereby hindering RNA Pol I transcription [199]. The H3K56ac modification is mediated by Asf1, a histone chaperone, and Rtt109, an acetyltransferase [181]. Both *asf1*Δ and *rtt109*Δ *S. cerevisiae* mutants are hypersensitive to rapamycin, which could suggest that TOR signaling influences histone modifications through a pathway that involves these proteins. However, further investigation is needed to fully elucidate this functional connection between TOR signaling, Asf1, and Rtt109 [199]. In addition to H3K56ac, TORC1 inhibition also leads to increased recruitment of histone deacetylase Rpd3 (Clr6 in fission yeast, HDAC2 in humans) to rDNA loci, leading to chromatin condensation and a condensed nucleolus and reduced RNA Pol I transcription in budding yeast [104]. Further evidence suggests that in *S. cerevisiae*, TORC1 components, such as Tor1 and Kog1, directly interact with 35S ribosomal DNA gene promoters in a manner sensitive to both rapamycin and starvation [200]. This interaction is crucial for the synthesis of 35S rRNA and, consequently, cell growth and implies that TORC1 may directly interact and promote heterochromatin formation, potentially through phosphorylating transcriptional machinery, which could cause rDNA repeat condensation [71,200]. Interestingly, *S. pombe* cells repress ribosomal gene transcription through a different mechanism [201]. Upon glucose starvation, *Sp*TORC1 dissociates from the rDNA region, leading to increased histone lysine 9 methylation and subsequent heterochromatin formation. This process is aided by the removal of the stress-responsive transcription factor Atf1 and the accumulation of the histone chaperone FACT [201]. This mechanism might be present in mammals containing the conserved heterochromatin factors Suv39H1 and HP1 [202], which are absent in budding yeast [203]. These findings in yeast suggest a chromatin-mediated mechanism through which TOR regulates nucleolar architecture, RNA Pol I distribution, and rRNA gene expression, adapting to changes in nutrient availability.

In yeast, inhibition of TORC1 leads to a rapid decline in rDNA transcription and subsequent rRNA production [204]. mTORC1 is also associated with rDNA promoter regions [104], though the biological significance of this interaction remains unclear. In mammals, the key regulators of RNA Pol I activity are TIF-1A and UBF (Upstream Binding Factor 1), analogous to Rrn3 and Hmo1 in yeast, respectively [179,205,206]. Treatment with rapamycin alters the phosphorylation states of TIF-1A and UBF, disrupting their interaction with RNA Pol I and consequently hindering rDNA transcription [94,207]. TFAM, the mammalian ortholog of Hmo1, is mainly characterized as a component of the mitochondrial transcription initiation complex, which has its own polymerase, mtRNAP or POLRMT [208,209]. Upon metabolic stress, this transcription factor forms a complex with FOXO3, a known mTORC2/AKT target, SIRT3, and POLRMT at mitochondrial DNA regulatory regions, thus promoting the expression of the mitochondrial genome and consequently increasing oxygen consumption [210]. While it remains unclear whether TFAM is a direct target of mTOR, like its yeast counterpart, TFAM translation is critically influenced by the 4E-BP pathway [211], suggesting that TFAM is at least indirectly influenced by TOR signaling. Together, these data highlight that TOR signaling and its downstream targets, direct or indirect, play an essential role in regulating RNA Pol I activity, which is central to ribosome biogenesis.

### 5.2. RNA Pol II

In yeast, about half of all RNA Pol II-mediated transcription is devoted to the production of ribosomal proteins to modulate ribosome biogenesis [186]. As previously discussed, TOR influences the expression of RNA Pol II-transcribed genes by affecting the nuclear/cytoplasmic localization of TFs through phosphorylation events. This portion of the review discusses genes of ribosomal proteins (RP) and ribosomal biogenesis (RiBi). In yeast, these genes are regulated by TOR via different mechanisms that directly or indirectly influence RNA Pol II activity. In contrast, mTORC1 regulates ribosomal protein gene expression mainly at the translational level [8]. mTORC1 promotes the translation of all ribosomal protein mRNAs [179]. In budding yeast, transcription factor Rap1 is a key player for both genes of ribosomal proteins and ribosomal biogenesis [212]. It binds to most ribosomal protein gene promoters and, thus, approximately half of all RNA Pol II transcripts [212]. Rap1 recruits a second transcription factor, Fhl1. Fhl1 is regulated by the coactivator Ifh1 and the corepressor Crf1 [71,213]. Under normal conditions, TORC1 keeps Crf1 within the cytoplasm via negative regulation of the kinase Yak1, preventing its interaction with Fhl1 and allowing the Rap1–Fhl1 complex to drive ribosomal protein gene expression [214]. Upon TORC1 inhibition, either through nutrient availability or otherwise rapamycin treatment, Yak1 can phosphorylate Crf1, and Crf1 can be transported to the nucleus and interfere with the Fhl1–Rap1 complex by interfering with Ifh1, leading to the downregulation of ribosomal protein gene expression [215]. In a recent study, CK2, another known TORC1 downstream effector, can also phosphorylate both Crf1 and Ifh1, vital in ribosome protein gene promoter binding [216].

Sfp1 is another factor involved in RNA Pol II transcription and is TOR-dependent [217]. It binds to promoters of ribosome biogenesis genes, leading to their upregulation. However, during stressful conditions, Sfp1 rapidly relocates from the nucleus to the cytoplasm, resulting in the downregulation of these genes. Additionally, Sfp1 serves as a negative regulator of Sch9, a key effector of TORC1 [217]. These interactions suggest a complex feedback mechanism or crosstalk that integrates TOR signaling, Sch9 activity, and RNA Pol II transcription, particularly affecting genes related to ribosomal proteins and ribosome biogenesis.

TOR signaling also regulates ribosome protein gene expression through Hmo1. Hmo1 is mainly known for its role in RNA Pol I transcription [191]; however, Hmo1 binds to most ribosomal protein gene promoters [218,219]. While the total effect of Hmo1 on ribosomal protein gene expression remains debated, the loss of Hmo1 negatively affects the expression of some ribosomal protein genes, suggesting that Hmo1 does play a role [219]. Hmo1 could be a potential linker between RNA Pol I and Pol II through TOR signaling as this protein has been reported to influence the transcription of genes of both polymerases, further supporting the idea that TOR signaling serves as a crucial hub for both transcription and translation and its intermediates. Other factors involved in TOR signaling and RNA Pol II transcription are Abf1, Sch9, and Sfp1 [71]. However, further studies are needed to fully understand the role that TOR signaling plays in ribosomal protein and ribosome biogenesis. Lastly, as with RNA Pol I, TORC1 plays a role in chromatin remodeling and histone modification during RNA Pol II-mediated transcription [71], but is yet to be fully characterized.

### 5.3. RNA Pol III

RNA Pol III synthesizes short RNAs over the DNA template through multiple rounds of transcriptional reinitiation [220]. RNA Pol III products include all tRNAs required for translation and 5S ribosomal RNA, crucial for ribosome biogenesis. Similar to RNA Pol I and Pol II, RNA Pol III transcription is disrupted upon rapamycin treatment and thus causes the downregulation of RNA Pol III-dependent genes [71,221], suggesting that TOR signaling, specifically TORC1, influences this polymerase. In *S. cerevisiae*, Maf1, a master regulator of RNA Pol III-mediated transcription, activity, and cellular localization, is regulated by phosphorylation [222,223]. TORC1 has been reported to be associated with Maf1 in vivo and phosphorylate Maf1 in vitro, and it is hypothesized that TORC1 is associated with 5S rDNA promoters [184]. Under permissive growth conditions, TORC1 directly phosphorylates Maf1, preventing Maf1 from accumulating in the nucleolus and thus interfering with Maf1 RNA Pol III association [184,224]. In this phosphorylated state, Maf1 is exported from the nucleus via Msn5 [184]. Under nutrient-poor conditions, Maf1 is dephosphorylated via PP4 [225] and is imported into the nucleus [184]. The dephosphorylated state of Maf1 correlates to increased Maf1 RNA Pol III interaction and RNA Pol III dissociation from DNA [184]. Other RNA Pol III machinery elements that are targets of TOR include Rpc53 and Bdp1 [71,226]. Rpc53 phosphorylation is TOR-dependent, and two downstream kinases of TOR, Kns1 and Mck1, are responsible for the phosphorylation of Rpc53 in nutrient-poor conditions [226]. In addition, Bdp1 is an essential subunit of the TFIIIB complex and is required for RNA Pol III recruitment onto promoters [185,227,228]. Multiple kinases, such as PKA, the TORC1-regulated kinase Sch9, and CK2, target Bdp1 [226], indicating an integrated regulatory framework for signaling events that control RNA Pol III transcription [224,229].

TOR signaling also regulates tRNA synthesis by controlling the activity of RNA Pol III, the enzyme responsible for tRNA transcription, primarily through regulating Maf1, which acts as a repressor of RNA Pol III, as previously discussed. When nutrient levels are high and *Sc*TORC1 is active, Maf1 is inhibited, allowing for increased tRNA production, while under nutrient deprivation, activated Maf1 suppresses tRNA synthesis [230]. In *S. cerevisiae*, TORC1 influences tRNA synthesis via the LAMMER/Cdc-like kinase Kns1 and CK2 [231]. When treated with rapamycin, Kns1 undergoes changes including increased expression, excessive phosphorylation, and relocation to the nucleus, where it then prepares the RNA polymerase III subunit Rpc53 for further phosphorylation by Mck1, a specific GSK-3 family kinase; this phosphorylation of Rpc53, combined with the dephosphorylation of the Maf1 repressor, ultimately results in the suppression of RNA polymerase III transcription, which is linked to cell growth inhibition [226]. CK2 is essential for activating RNA polymerase III activity as cells shift from repressive to favorable growth conditions. This activation occurs through the phosphorylation of Maf1, which promotes its dissociation from the RNA polymerase III complex [231]. The CK2 regulatory subunit, Ckb1, is phosphorylated by Kns1 under various stress conditions, including treatment with rapamycin [232]. This phosphorylation decreases CK2 occupancy at tRNA genes, thereby repressing RNA Pol III activity. Moreover, a genetic screening of rapamycin-sensitive *S. pombe* mutants revealed that genes involved in tRNA modification were highly enriched, indicating a significant link between tRNA modification and TORC1 activity [233].

In mammals, mTORC1 inhibition downregulates RNA Pol III transcription [234]. Like in yeast, mammalian RNA Pol III regulation depends on the MAF1 repressor. mTORC1 inhibition causes MAF1 dephosphorylation [235,236]. Interestingly, mTORC1 directly phosphorylates and regulates MAF1 [233]. Additionally, mTORC1 is associated with RNA Pol III genes [103,104], predominantly within tRNA genes [103]. It has been suggested that TFIIIC recruits mTORC1 to these DNA regions, where mTORC1 then phosphorylates and displaces MAF1 from chromatin, facilitating transcription [103]. Thus, the mechanism by which mTORC1 inhibition attenuates RNA Pol III transcription is at least partially conserved across species.

### 5.4. TOR Pathways Might Mediate Cross-Regulation of These Three RNA Pols

In yeast, regulating three RNA Pols through the TOR pathway, particularly via the Sch9 kinase, involves complex interactions and phosphorylation events. Sch9 indirectly affects transcription by phosphorylating the Maf1 and Bdp1 proteins and modulating the expression of the ribosomal protein and ribosomal biogenesis genes by targeting Dot6, Tod6, and Stb3 [71]. Although Sch9 seems to influence RNA Pol I recruitment and transcription [221], the specifics of this interaction are not fully understood. Additionally, shared subunits of the three RNA Pols, such as Rpb5, have been identified as phosphorylation targets, potentially through TOR signaling. This includes interactions with Bud27 (and its human ortholog URI), which is essential for the assembly and function of the RNA Pols and may be a coordination point for their activity [237,238]. Furthermore, the universal transcription factor TBP, regulated by TOR via CK2 phosphorylation, indicates another layer of control over RNA Pol transcription [239,240]. The demethylase Rph1, which interacts with TOR, modulates RNA Pol I and II under nutrient stress [241], pointing to a broader regulatory role of TOR over transcription related to cell survival. Intriguingly, chromatin remodeling factors like Chromatin Structure Remodeling (RSC) complex are also implicated in the TOR-dependent regulation of transcription [242], suggesting a multifaceted control mechanism over ribosome biogenesis by the TOR pathway.

The coordination of the three RNA Pols by mTORC1 in mammals is implicated but remains poorly understood. MAF1 is considered a key coordinator, as it regulates not just RNA Pol III but also RNA Pols I and II [243]. Other potential coordinators include universal factors like c-Myc, p53, and TBP, which are involved in the transcription mediated by all three RNA Pols [71]. Additionally, URI, the human counterpart of Bud27 in budding yeast, is suggested to play a coordinating role [238]. URI is linked to both the mTOR pathway and gene expression, potentially through its interactions with the common subunit RPB5 of RNA polymerases [237].

## 6. Conclusions

Coordinating transcription within the nucleus and protein synthesis in the cytoplasm presents a complex challenge in eukaryotic gene expression. Despite the critical role of these processes, the mechanisms that link them are not fully elucidated. The TOR signaling pathway is essential in integrating nutrient signals and orchestrating a well-coordinated response across transcriptional and translational levels to maintain cellular homeostasis, adaptation, and survival across varying nutrient conditions.

TOR signaling dynamically regulates cellular responses based on nutritional status, balancing growth and maintenance to optimize survival. It achieves this through its two complexes, TORC1 and TORC2. These complexes coordinate the expression of crucial transcription factors, regulate the activity of RNA polymerases I, II, and III, and influence ribosome biogenesis and protein translation. This ensures appropriate communication between the transcriptional activities in the nucleus and translation processes in the cytoplasm. Furthermore, the role of TOR signaling extends beyond its well-established functions in transcription and translation. The pathway also plays roles in RNA processing and exporting, highlighting its complex impact on gene expression. In addition to managing regular growth and metabolic activities under standard conditions, TOR signaling is critical in mounting an appropriate stress response by interacting with the MAPK signaling and the ISR. This review highlights the importance of TOR as a central facilitator in gene expression, integrating transcription, translation, and their intermediaries into a cohesive network that is essential for cellular viability.

Further research is essential to elucidate the TOR-mediated interplay between transcription and translation fully. The complexity of TOR signaling is considerable, featuring multiple feedback and feed-forward loops, competing protein interactions, and extensive crosstalk with numerous other signaling pathways [88]. Additional cellular pathways, such as the MAPK/ERK, Wnt/β-catenin, and NF-κB pathways, also play roles in coordinating transcription and translation [34,84,85]. While it is recognized that TOR engages in crosstalk with many of these signaling pathways, the precise nature of these interactions remains poorly defined. More detailed exploration is required to fully understand their cooperative or antagonistic dynamics.

With advancements in artificial intelligence technology, computational modeling has become an invaluable tool for investigating complex network properties. This approach is increasingly instrumental in predicting and synthesizing accurate and comprehensive biological knowledge, offering new insights into the regulatory mechanisms underlying cellular functions. This convergence of technology and biology promises to enhance our understanding of how intricate signaling networks, such as those involving TOR, coordinately regulate transcription and translation.

## Figures and Tables

**Table 1:** Components, effectors, and orthologs of TOR complexes.

	mTOR	*Sc*TOR	*Sp*TOR
Complexes	mTORC1/mTORC2	TORC1/TORC2	TORC1/TORC2
Components of (m)TORC1	mTOR, RAPTOR, mLST8	Tor1 or Tor2, Kog1, Lst8, Tco89	Tor2, Mip1, Wat1, Tco89
Components of (m)TORC2	mTOR, RICTOR, mSIN1, mLST8	Tor2, Avo1, Avo2, Avo3	Tor1, Ste20, Sin1, Wat1, Bit61
Upstream Effectors of (m)TORC1	RAGULATOR complex GATOR1/2 complexes	EGO ternary complex SEACIT/SEACAT complexes	- GATOR1/SEACIT and GATOR2/SEACAT
